# ENTORHINAL CORTEX THICKNESS IS ASSOCIATED WITH FINANCIAL EXPLOITATION VULNERABILITY IN OLDER ADULTS

**DOI:** 10.1093/geroni/igae098.3618

**Published:** 2024-12-31

**Authors:** Laura Fenton, Lauren Salminen, Aaron Lim, Gali Weissberger, Annie Nguyen, Jenna Axelrod, Daisy Noriega-Makarskyy, Duke Han

**Affiliations:** University of Southern California, California, United States; University of Southern California, Los Angeles, California, United States; University of Southern California, Los Angeles, California, United States; Bar-Ilan University, Ramat Gan, HaMerkaz, Israel; University of California San Diego, La Jolla, California, United States; University of Southern California, Los Angeles, California, United States; University of Southern California, Los Angeles, California, United States; University of Southern California, Los Angeles, California, United States

## Abstract

Research suggests that increased financial exploitation vulnerability (FEV) due to declining decision making may be an early behavioral manifestation of brain changes occurring in preclinical Alzheimer’s disease (AD). One of the earliest documented brain changes during this preclinical phase is neurodegeneration of the entorhinal cortex. The objective of the current study was to examine the association between a measure of FEV and thickness of the entorhinal cortex in 97 older adults without cognitive impairment (mean age (SD) = 68.72 (7.45); mean education (SD) = 16.52 (2.14); 76% female; 76% White). We also investigated FEV associations with frontal regions typically associated with decision making (e.g., dorsolateral and ventromedial prefrontal cortices). Exploratory analyses investigated the interactive effect of age and cortical thickness on FEV. Results showed that greater FEV was associated with significantly lower entorhinal cortex thickness (B = -0.05, SE = 0.02, p = 0.04), but not with thickness in frontal regions. There was a significant interaction between age and entorhinal cortex thickness on FEV, whereby lower entorhinal cortex thickness was associated with greater FEV in older participants (B = -0.01, SE = 0.003, p = 0.04). When the group was split according to age using a median split (70+; < 70 years old), lower entorhinal cortex thickness was associated with greater FEV only in the older group (B = -0.10, SE = 0.04, p = 0.01). Collectively these findings support the notion that FEV may serve as an early behavioral manifestation of preclinical AD before noticeable cognitive impairment.

